# Topography of Pericholecystic Adhesions as a Determinant of Operative Difficulty During Laparoscopic Cholecystectomy Using the Parkland Grading Scale: A Prospective Observational Study

**DOI:** 10.7759/cureus.105957

**Published:** 2026-03-27

**Authors:** Aishwarya Priyadarsani Bhuyan, Mukul Singh, Gaurav Gupta, Ravi Gupta, Dharmendra Kumar Pipal, Harikesh Yadav, Rajnish Kumar, Shahnawaz Ahmed Chowdhary, Manish Kumar, Aditya Gaurav, Swati Prasad

**Affiliations:** 1 General Surgery, All India Institute of Medical Sciences, Gorakhpur, IND

**Keywords:** adhesions, gallbladder, laparoscopic cholecystectomy, operative difficulty, parkland grading

## Abstract

Background

Adhesions around the gallbladder are a major cause of difficulty during laparoscopic cholecystectomy (LC). While the presence of adhesions has been widely discussed, the exact location of these adhesions and their influence on operative difficulty have rarely been studied.

Aim

To evaluate whether the anatomical location of pericholecystic adhesions (neck, body, whole gallbladder, or absent) correlates with operative difficulty as assessed by the Parkland Grading Scale (PGS).

Methods

A prospective observational study of 100 patients undergoing LC was conducted. During the initial laparoscopic view, the location of adhesions was documented and categorized into four groups. These were correlated with Parkland grades recorded intraoperatively.

Results

Adhesions were absent in 59 cases (59%), predominantly corresponding to Parkland Grade 1. Neck adhesions were present in 23 cases (23%) and showed a strong association with higher Parkland grades. Whole gallbladder adhesions were observed in eight cases (8%) and were consistently associated with Grades 4 and 5. Body adhesions were identified in 10 cases (10%) and demonstrated an intermediate association with operative difficulty.

Conclusions

The location of adhesions is a significant determinant of operative difficulty. Neck and whole gallbladder adhesions predict higher operative complexity and should alert the surgeon to anticipate difficult dissection early.

## Introduction

Laparoscopic cholecystectomy is currently viewed as the gold standard in the surgical treatment of symptomatic gallstone disease and is one of the most frequent types of surgeries done in the world today [1]. Since it was introduced in the late 1980s, laparoscopic cholecystectomy has predominantly replaced open cholecystectomy due to the advantages of decreased pain after surgery, shorter length of time in the hospital after abdominal surgery, and a rapid return to full activity [2]. While this procedure is regarded as routine, the level of difficulty during surgery can vary based on multiple patient factors and disease factors [3]. The more difficult a case is, the longer the amount of time the operation will run, and the higher the number of injuries to the bile duct and the higher the chance of conversion to open surgery will occur. Each of these variables can have an impact on patient outcomes.

One of the leading contributors to the increased difficulty of laparoscopic cholecystectomy is peri-cholecystic adhesion tissue [4]. Adhesion tissue usually occurs because of a ruptured or recurring episodes of infection, causing acute or chronic cholecystitis, resulting in adhesions between the gallbladder and adjacent tissues such as the omentum, duodenum, and colon [5]. This adhesion tissue can obscure important landmarks that are necessary to perform a safe dissection.

For many years, adhesions have been evaluated by using a binary classification of present or absent. However, this system does not accurately capture the importance of these adhesions to the surgical procedure [6]. The anatomical location of the adhesions is an important factor in determining the level of surgical complexity for laparoscopic cholecystectomy. Adhesions to the gallbladder neck and hepatocystic triangle are critical as these will alter the normal appearance of the biliary system and are often necessary to attain the critical view of safety for the gallbladder and increase the risk of injury to the biliary system [7-9].

The Parkland Grading Scale (PGS) is a grading system for evaluating the visual findings of the gallbladder based on inflammation present on the initial laparoscopic view of the gallbladder based on the degree to which inflammation is present, the presence of adhesion tissue, and distortions in the normal anatomy relating to the gallbladder. Prior studies have shown that higher grades within the PGS correlate with longer time required for surgery and a higher rate of conversion to open procedures [10,11].

To date, there is limited literature evaluating the topographic distribution of pericholecystic adhesions and their impact on operative difficulty using a standardized intraoperative grading system. Therefore, this study aimed to evaluate the relationship between the anatomical location of pericholecystic adhesions and operative difficulty using the PGS, with the objective of improving early intraoperative prediction of surgical complexity and guiding operative decision-making during laparoscopic cholecystectomy.

## Materials and methods

Study design and setting

This prospective observational study was conducted in the Department of General Surgery at AIIMS Gorakhpur from February 2024 to August 2025. A total of 100 consecutive patients with symptomatic gallstone disease undergoing elective laparoscopic cholecystectomy were included. All procedures were performed by surgeons experienced in laparoscopic cholecystectomy, following standardized institutional protocols. Intraoperative findings were prospectively documented. Ethical approval was obtained from the Institutional Human Ethics Committee, AIIMS Gorakhpur (IHEC Ref No.: IHEC/AIIMS-GKP/BMR/233/2024).

Selection Criteria

Patients aged 18 years and above undergoing elective laparoscopic cholecystectomy for symptomatic gallstone disease were included. Patients were excluded if they were pregnant, undergoing concurrent surgical procedures, or did not provide informed consent.

Sample Size Calculation

The sample size was determined based on feasibility and expected case volume during the study period. A sample of 100 patients was considered adequate to evaluate the association between adhesion location and Parkland grading using categorical statistical analysis, assuming a significance level (α) of 0.05 and a statistical power of 80%.

Data sources and variables

Preoperative data, including demographic details, clinical history, biochemical parameters, and ultrasonographic findings, were recorded. Intraoperative assessment was performed after placement of all laparoscopic ports and prior to any dissection to minimize bias. Adhesions were defined as omental or visceral attachments involving the gallbladder identified on the initial laparoscopic view. Based on anatomical location, adhesions were categorized into four groups: no adhesions, adhesions involving the gallbladder neck, adhesions involving the body, and adhesions involving the entire gallbladder. Operative difficulty was assessed using the PGS, an intraoperative grading system originally described by Madni et al. [12] and subsequently validated in later studies [10]. Grading was performed by the operating surgeon according to predefined criteria. In cases of uncertainty, intraoperative consensus was obtained among the surgical team to ensure consistency in assessment.

Statistical analysis

Data were analyzed using Statistical Package for the Social Sciences (IBM SPSS Statistics, Version 31.0, IBM Corp., Armonk, NY, USA). Descriptive statistics were used to summarize patient characteristics and study variables. Categorical variables were expressed as frequencies and percentages, while continuous variables were summarized using median and interquartile range. The association between adhesion location and Parkland Grading Scale categories was evaluated using Pearson’s chi-square test. A p-value of <0.05 was considered statistically significant. As this was an exploratory analysis, no adjustment for potential confounders was performed.

## Results

The study population's anthropometric and demographic parameters are displayed in Table 1. There were 100 patients in all, most of whom were female. Participants were mostly between the ages of 30 and 49. Most of them weighed between 50 and 70 kg and were between 140 and 160 cm tall. Obesity was less common than overweight, which was the most common BMI category, followed by normal.

**Table 1:** Demographic characteristics of the study population (N=100). Values presented as frequency (n) and percentage (%). BMI: body mass index.

Variable	Category	Frequency (n)	Percentage (%)
Gender	Female	86	86.0
	Male	14	14.0
Age (years)	10-19	2	2.0
	20-29	24	24.0
	30-39	32	32.0
	40-49	29	29.0
	50-59	10	10.0
	60-69	3	3.0
Height (cm)	130-140	3	3.0
	140-150	29	29.0
	150-160	50	50.0
	160-170	16	16.0
	170-180	2	2.0
Weight (kg)	40-50	14	14.0
	50-60	27	27.0
	60-70	38	38.0
	70-80	15	15.0
	80-90	5	5.0
	90-100	1	1.0
BMI Category	Normal	38	38.0
	Overweight	45	45.0
	Obese Class I	15	15.0
	Obese Class II	2	2.0

The research population's preoperative clinical characteristics are displayed in Table 2. The majority of individuals had never been hospitalised for pancreatitis or acute cholecystitis. Acute cholecystitis was more prevalent than acute biliary pancreatitis among patients who had previously been hospitalised. Most of the patients had never had abdominal surgery before.

**Table 2:** Preoperative clinical characteristics (N=100). Values presented as frequency (n) and percentage (%).

Pre-operative History	Category	Frequency (n)	Percentage (%)
Previous hospitalization for acute cholecystitis or pancreatitis	No	89	89.0
	Yes	11	11.0
Type of previous episode	Acute biliary pancreatitis	4	4.0
	Acute cholecystitis	7	7.0
History of previous abdominal surgery	No	68	68.0
	Yes	32	32.0

The research population's ultrasonographic results are displayed in Table 3. The gallbladder wall thickness was normal in the majority of patients, and no pericholecystic fluid accumulation was found. In a small percentage of instances, gallbladder sludge was seen. Most patients had normal liver size, with a lesser percentage showing signs of hepatomegaly.

**Table 3:** Ultrasonographic parameters (N=100). Values presented as frequency (n) and percentage (%).

Parameter	Category	Frequency (n)	Percentage (%)
Gallbladder wall thickness	Normal	96	96.0
	Diffuse wall thickening (4 mm)	2	2.0
	Contracted gallbladder	1	1.0
	Focal ring-like thickening	1	1.0
Pericholecystic fluid collection	No	100	100.0
Gallbladder sludge	No	90	90.0
	Yes	10	10.0
Condition of liver	Normal size	81	81.0
	Hepatomegaly	19	19.0

Preoperative biochemical parameters are summarized in Table 4. Median haemoglobin values suggested mild anaemia, while total leukocyte counts were within normal limits. Liver function parameters were largely within physiological ranges.

**Table 4:** Biochemical parameters. Preoperative biochemical parameters are expressed as median with interquartile range (IQR: 25th-75th percentile) due to non-normal distribution of data. SGOT: serum glutamic oxaloacetic transaminase; SGPT: serum glutamic pyruvic transaminase; ALP: alkaline phosphatase.

Parameter	Median (IQR)
Haemoglobin (g/dL)	11.4 (10.8-12.2)
Total leukocyte count (×10⁹/L)	7.12 (6.30-8.40)
SGOT (IU/L)	32.34 (26.0-39.5)
SGPT (IU/L)	31.95 (25.5-38.0)
ALP (IU/L)	85.0 (72.0-98.0)
Total bilirubin (mg/dL)	0.525 (0.40-0.68)
Direct bilirubin (mg/dL)	0.180 (0.12-0.25)
Indirect bilirubin (mg/dL)	0.260 (0.18-0.36)

The distribution of adhesion locations based on the Parkland Grading Scale is displayed in Table 5. While neck adhesions were seen in intermediate and higher grades, the absence of adhesions was primarily linked to lower Parkland grades. The majority of whole gallbladder adhesions were found in higher Parkland grades. Adhesion position and Parkland grading were shown to be statistically significantly correlated (χ²=66.443, df=12, p<0.001). A progressive increase in Parkland grade was observed with more extensive adhesion involvement, particularly in cases involving the gallbladder neck and entire gallbladder.

**Table 5:** Association between adhesion location and Parkland Grading Scale grades (N=100). Values presented as frequency (n) and percentage (%). Pearson’s chi-square test demonstrated a significant association between adhesion location and Parkland grades (χ²=66.443, df=12, p<0.001).

Location of Adhesions	Grade 1	Grade 2	Grade 3	Grade 4	Grade 5	Total (n)	Percentage (%)
Body	1	3	3	2	1	10	10.0
Neck	13	5	2	1	2	23	23.0
No adhesions	51	5	3	0	0	59	59.0
Whole gallbladder	0	0	2	2	4	8	8.0
Total	65	13	10	5	7	100	100.0

## Discussion

The present study demonstrates that the topographical distribution of pericholecystic adhesions is significantly associated with operative difficulty during laparoscopic cholecystectomy. The results show that not all adhesions have an identical effect on surgical difficulties, despite the fact that adhesions are commonly acknowledged as contributors to technical complexity. The importance of evaluating the anatomical location of adhesions rather than just documenting whether there is an adhesion present was reemphasised.

The PGS, first introduced and validated by Madni et al. in 2018, provides an objective intraoperative assessment based on the first laparoscopic view of the gallbladder. Previous studies have shown that higher Parkland grades are associated with increased operative difficulty, longer operative time, and higher rates of conversion to open surgery. This study showed that the majority of patients without pericholecystic adhesions were assigned a low (or grade I) level on the PGS, which supports the theory that simple dissection and less complicated operations are associated with less inflammatory reaction [12].

In addition, there was a statistically significant correlation between higher Parkland grades (grades III to V) and the presence of pericholecystic adhesions around the neck of the gallbladder. The anatomical relationship between the neck of the gallbladder and the hepatocystic (Calot’s) triangle, which is the key area to find the cystic artery and cystic duct. This may be attributed to recurrent episodes of inflammation caused by transient obstruction of the cystic duct, leading to preferential adhesion formation in this region. The presence of pericholecystic adhesions distorts the anatomy of the hepatocystic triangle, which adversely affects the technical achievement of the critical view of safety. These findings emphasize that adhesions at the gallbladder neck may serve as an early intraoperative indicator of increased technical difficulty.

The data presented is consistent with the results from research performed by Strasberg et al., which emphasized the importance of achieving the critical view of safety during laparoscopic cholecystectomy and that anatomical structures within Calot's triangle are frequently misinterpreted, leading to injury to the common bile duct [13]. Dense adhesions in the area can lead to increased surgical complexities and difficulties in identifying anatomical structures. The Tokyo Guidelines, published in 2018, also indicated that significant local inflammation around the hepatocystic triangle has been identified as a major cause of increased surgical complexity in the cholecystectomy [14].

Adhesions covering the entire gallbladder were primarily associated with Parkland grades IV and V, which identify the possibility of severe anatomical distortion and increased inflammatory disease. The majority of cases require significant dissection to remove the adhesions prior to completing the dissection safely. Similarly, Sugrue et al. demonstrated that patients with large dense adhesions, especially among constricted gallbladders, demonstrate a greater need for conversion to open procedures and difficulty completing their operations [15]. Such cases may necessitate advanced dissection strategies or consideration of bailout procedures.

Conversely, most cases associated with intermediate Parkland grades were only associated with adhesions located within the body of the gallbladder. Although these adhesions do not typically compromise the anatomy of the hepatocystic triangle at that moment, they can increase the length of time for exposure as well as the length of time for dissecting the gallbladder from the liver. Previous studies have also illustrated that the instance of having adhesions alone is not as strongly correlated to surgical complexity as the distortion of key biliary anatomy [11,13].

These findings have important clinical implications. Early identification of adhesion topography during a laparoscopic initial view may allow early prediction of operative complexity and help guide intraoperative decision-making. By early identification of high-risk anatomy during the operation, as indicated by the Tokyo Guidelines 2018, appropriate dissection techniques can be used, senior assistance can be sought, or consideration of alternative surgical techniques can be performed, such as partial cholecystectomy in complex situations [16].

Furthermore, the data also support the PGS as a useful intraoperative assessment tool that is objective and easily reproduced. The Parkland Grading Scale provides a structured methodology for predicting problems during the operative procedure and helps improve operative safety by correlating intraoperative anatomical findings with the level of surgical difficulty.

A key limitation of this study is the absence of operative outcome measures such as operative time, conversion to open surgery, and postoperative complications. Therefore, while a significant association with intraoperative difficulty was demonstrated, the direct impact on clinical outcomes could not be established. Future studies incorporating these parameters are required to better define the clinical applicability of adhesion topography.

Strengths and limitations

This study utilized the standardized PGS to prospectively assess adhesion topography, enabling objective intraoperative evaluation and correlation with operative difficulty. Adhesions were documented at the first laparoscopic view, which decreased intraoperative manipulation bias. However, generalisability may be limited by the study's single-center design and small sample size. Furthermore, despite the implementation of a standardized system, grading was dependent on the surgeon and might be influenced by observer variability. Additionally, the absence of operative outcome measures limits the ability to directly correlate these findings with clinical outcomes. To confirm these results, more multicenter trials with bigger cohorts and correlation with operative outcomes are necessary. 

## Conclusions

During laparoscopic cholecystectomy, adhesion topography appears to be an important factor influencing operative difficulty. While whole gallbladder adhesions are associated with advanced inflammatory changes and higher Parkland grades, adhesions involving the gallbladder neck and hepatocystic triangle are also associated with increased operative complexity. Adhesions limited to the gallbladder body are generally associated with intermediate levels of difficulty. Identification of adhesion location during the initial laparoscopic evaluation may help anticipate operative difficulty and support intraoperative decision-making; however, further studies incorporating operative outcomes are needed to confirm its clinical applicability.
